# Supplementary figures and images for: The Organization of the Golgi Structures during Drosophila Male Meiosis Requires the Citrate Lyase ATPCL
Source: Int J Mol Sci. 2021 May 27;22(11):5745. doi: 10.3390/ijms22115745 (PMC8199154; doi:10.3390/ijms22115745)

*DmATPCL*<sup>01466</sup>/  
*Df(2R)Exel7138*

*Oregon R*

LVA

DAPI

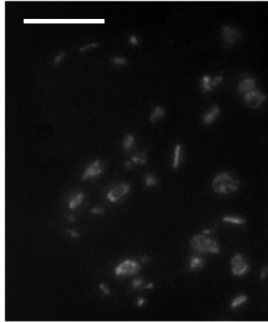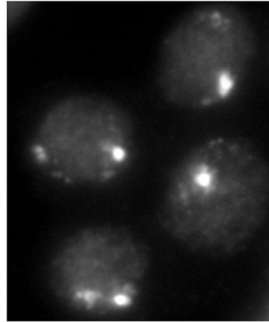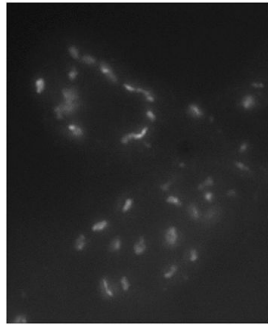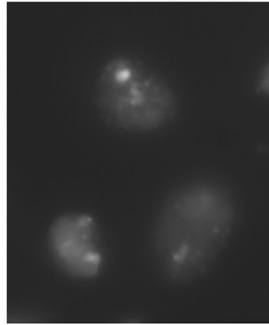

Supplement: Supplementary file 1 [file ijms-22-05745-s001.zip › ijms-1231641-supplementary.pdf]
